# Omics-AD—A multimodal biomarker study on cognitive decline and neuropsychiatric symptoms: Design and cohort characteristics

**DOI:** 10.1177/13872877251401159

**Published:** 2025-12-04

**Authors:** Miriam Rabl, Leonardo Zullo, Jelena Wehrli, Karolin Hössel, Piotr Lewczuk, Iliya Petkov Peyneshki, Erich Seifritz, Stefan Klöppel, Armin von Gunten, Julius Popp

**Affiliations:** 1 Department of Adult Psychiatry and Psychotherapy, Psychiatric University Hospital Zurich and University of Zurich, Zurich, Switzerland; 2 Old Age Psychiatry Service, Department of Psychiatry, Lausanne University Hospital, Lausanne, Switzerland; 3 Leenaards Memory Clinic, Lausanne University Hospital, Lausanne, Switzerland; 4 Department of Psychiatry and Psychotherapy, Universitätsklinikum Erlangen and Friedrich-Alexander Universität Erlangen-Nürnberg, Erlangen, Germany; 5 Department of Neurodegeneration Diagnostics, Medical University of Białystok, Białystok, Poland; 6 University Hospital of Old Age Psychiatry and Psychotherapy, University of Bern, Bern, Switzerland

**Keywords:** Alzheimer's disease, multi-omics, biomarker, neuropsychiatric symptoms, subjective cognitive decline

## Abstract

**Background:**

Alzheimer's disease (AD) clinically manifests in cognitive decline and frequent neuropsychiatric symptoms (NPS).

**Objective:**

The Omics-AD study's scope is to perform an in-depth multi-modal and longitudinal characterization of people with early AD to a) better understand pathophysiological changes of AD and b) identify new biomarkers for AD and AD-related clinical manifestation and progression, with a focus on NPS.

**Methods:**

Participants in this prospective study were recruited at four Swiss memory-clinics. Comprehensive cognitive and neuropsychiatric assessments were performed at baseline and follow-up. Paired blood and cerebrospinal fluid (CSF) samples along with structural MRI were obtained at baseline. Established CSF AD biomarkers were analyzed. Untargeted omics and targeted molecular analyses will be performed and integrated in multi-modal, multi-omics data analysis.

**Results:**

We included 456 participants (mean age 71.2 years, 55.1% female), of which 48.5% were cognitively unimpaired (with no cognitive complains, NC, or with subjective cognitive decline, SCD) and 51.5% cognitively impaired (mild cognitive impairment, MCI, or mild clinical AD dementia). Half of the participants presented with NPS as measured by the Neuropsychiatric Inventory Questionnaire (48.5%) or the Mild Behavioral Impairment Checklist (52.7%). The most common symptoms were irritability (18%) and depression (17%). In total, 41.0% (n = 155) of participants were amyloid positive (20.6% of CN, 21.7% of SCD, 55.4% of MCI, and 72.4% of clinical AD dementia).

**Conclusions:**

This multi-centric well-characterized cohort allows for single- and multi-omics analyses to investigate in depth molecular and biological pathway alterations in AD and their relationships with clinical manifestation and progression, with a particular focus on NPS.

## Introduction

Alzheimer's disease (AD) is the most common cause of dementia.^1^ In addition to cognitive and functional decline that clinically characterize the disease, neuropsychiatric symptoms (NPS) are very common, may emerge in early stages, and substantially contribute to reduced quality of life and accelerated disease progression.^2–4^ The pathophysiological events of AD begin more than two decades before the clinical onset of dementia.^5^ Amyloid aggregation (abnormal amyloid-β (Aβ) protein metabolism resulting in amyloid plaque deposition) together with tauopathy (hyperphosphorylation of tau and neurofibrillary tangles) constitute the core pathological hallmarks of AD.^6,7^ In addition, neuronal injury (neuronal dysfunction, cell death, and brain atrophy) and neuroinflammation play important roles in the pathogenesis of AD. Cerebrospinal fluid (CSF) levels of amyloid-β 42 (Aβ_42_), tau phosphorylated at threonine 181 (pTau181), and total tau (tTau) are the best validated biofluid biomarkers of amyloid pathology, tau pathology, and neurodegeneration, respectively. More recently, new biomarkers are increasingly being validated, including blood-based biomarkers such as pTau 217 and pTau213 for tauopathy, neurofilament light chain (NfL) for neuronal injury, and glial fibrillary acidic protein (GFAP) for neuroinflammation.^7^

However, several other molecular and pathway alterations at both the central nervous system and systemic levels have been reported in AD.^8–11^ The interindividual variations, complex interrelations, and relevance of these alterations for clinical manifestation and disease progression are still poorly understood. A better understanding of these processes could help discover and validate biomarkers to improve early and differential diagnosis, as well as the prediction of clinical disease progression. Finally, these pathway alterations could represent personalized intervention targets for prevention and treatment.

Investigating pathophysiological changes in AD requires comprehensive assessments. Over the past decade, “-omics” approaches have advanced significantly,^12^ enabling the simultaneous measurement of thousands of molecules and providing sensitive readouts of biological function and disease-specific molecular fingerprints.^13,14^ This development has accelerated the understanding of pathophysiological mechanisms underlying complex diseases, including AD.^15–17^ In addition to identifying altered biofluid molecular profiles as potential diagnostic biomarkers in AD, single omics and multi-omics approaches may also allow for the exploration and investigation of pathway alterations in greater depth. However, these untargeted approaches need to be complemented with hypothesis-driven targeted approaches to validate and extend the findings and allow for clinical translation.^18^

The biological mechanisms involved in NPS have not been fully characterized yet. While previous studies have examined associations between NPS and individual modalities such as imaging, molecular, or clinical data, few studies have integrated more than two data types.^19^ Moreover, unbiased molecular omics approaches (e.g., untargeted proteomics or metabolomics) and their integration into multi-omics models are largely lacking,^20^ limiting our understanding of the pathomechanisms underlying NPS.

We aimed to perform a multi-omics characterization of a large, multi-centric, brain aging cohort with a focus on early and preclinical AD in a memory clinic setting. The main goals were to a) better understand the pathophysiological changes in AD and related neurocognitive and NPS, including molecular phenotyping, and to b) identify and validate new biomarker candidates for AD and AD-related outcomes such as cognitive decline or the persistence of NPS. As a secondary goal, we wanted to identify easily accessible blood biomarkers for potential clinical application. The primary intention of this work is to present the participants’ baseline characteristics, describe the study design, and provide a reference framework for future hypothesis-driven analyses using the multimodal Omics-AD dataset.

## Methods

### Design and study population

The Swiss Omics-AD study is a four-center prospective longitudinal cohort study. It includes participants with different cognitive stages ranging from cognitively healthy through mild cognitive impairment to mild dementia with or without underlying AD pathology, with a focus on early and preclinical AD patients.

The participants were community-dwelling older people recruited at memory clinics among patients seeking evaluation of their cognitive complaints or through advertisements. Recruitment for the first center (Lausanne-1) ran between 2013 and 2017. Upon completion and receipt of new funding the same protocol was applied to continue the study at three additional centers. The recruitment periods for the single centers were as follows: Zurich 2021–2025, Bern 2022–2023, and Lausanne-2 2023–2025.

To fulfill the inclusion criteria, participants had to be older than 60 years and community-dwelling. The exclusion criteria were moderate or severe dementia without the capacity to provie informed consent, poor language skills, contraindications for MRI or lumbar puncture, other known or suspected neurodegenerative disorders such as Parkinson's disease or Lewy body disease, or an unstable medical condition or medication that may interfere with cognition or severely affect the metabolism (e.g., autoimmune disorders, unstable diabetes, substance dependency, and chemotherapy).

The clinical diagnosis was made by a consensus conference, which included physicians and neuropsychologists experienced in the field, on the basis of available clinical and neuropsychological assessments retrieved at each visit. All centers used state-of-the-art criteria for the diagnosis. We included cognitively unimpaired participants (normal cognition, NC), participants with subjective cognitive decline (SCD), mild cognitive impairment (MCI), and mild clinical AD dementia. Participants classified as NC had no objective cognitive impairment, defined by a performance above −1.5 z-scores on cognitive tasks of the neuropsychological test battery (memory, attention, executive functioning, language, or visuo-construction). In addition, they had a Clinical Dementia Rating (CDR) score of 0 and reported no memory complaints on the Subjective Cognitive Decline Interview (SCD-I, question 1).^21^ Participants with SCD also had no objective cognitive impairment with a CDR score of 0, but self-reported memory complaints (SCD-I, question 1^21^) at inclusion.^22^ MCI was diagnosed via consensus criteria for MCI.^23^ Participants had objective cognitive impairment in at least one cognitive domain, defined by a performance below −1.5 z-scores on cognitive tasks of the neuropsychological test battery (memory, attention, executive functioning, language, or visuo-construction). They also had a CDR score of 0.5 and no or only minimal impairment of activities of daily living (ADL), as assessed by the instrumental activities of daily living (IADL).^24^ The diagnosis of clinical AD dementia was defined based on the McKhann criteria for dementia due to AD, with a CDR score of ≥1 and impairment in the IADL.^25^ We included only participants with the capacity to provide informed consent at baseline. At the time of clinical diagnosis, all study personnel were blinded to the presence or absence of CSF core AD biomarkers.

**Figure 1.**
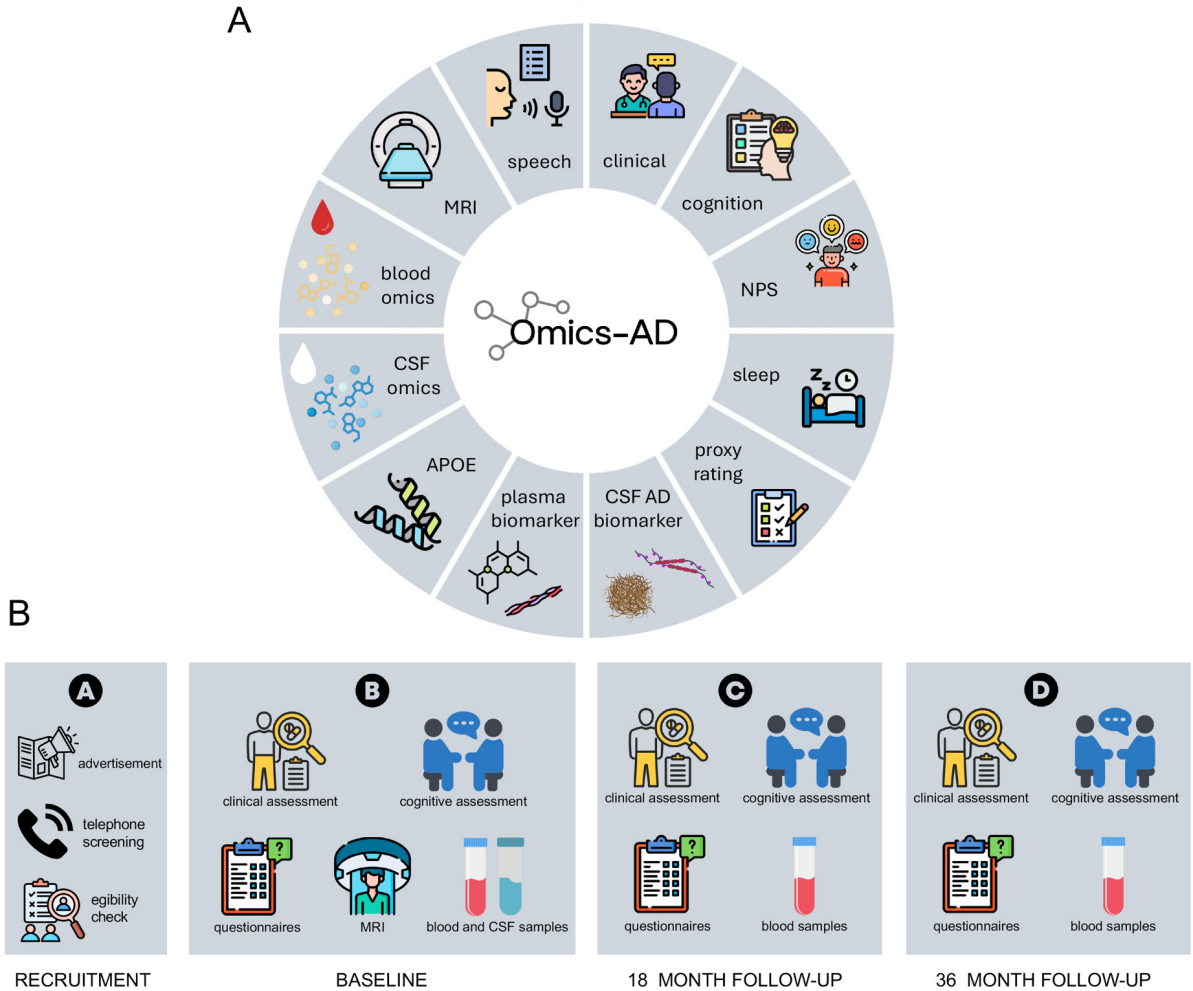
Overview of assessments performed within the Omics-AD study.

An overview of the assessments performed within the Omics-AD study is shown in Figure 1A. All the participants underwent comprehensive clinical and neuropsychological evaluations by trained and memory clinic experienced personnel. All tests and scales used in this study are routinely and widely used in memory clinics and have been validated in the field. The enrolled participants were invited to at least two follow-up visits after 18 and 36 months (see Figure 1B). A detailed list of the performed clinical assessments is provided in Table 1.

**Table 1.** Detailed list of all performed assessments within the Omics-AD study.

Category	Domain	Name of test or questionnaire
Clinical assessment	Anamnesis	General anamnesis and evaluation
		Medical history
		Family history
		Substance use
		Physical and neurological examination
		History of Covid-19 (since 2021)
		Current medications including supplements
	Physical measurement	Weight, Height, BMI
		Blood pressure, heart rate
	Global rating	CDR and CDR-SB
Cognitive assessment	Global cognition	MoCA, MMSE alternatively
	Memory	CERAD-NAB: verbal learning, recall & recognition, and figural recall
	Attention	CERAD-NAB: TMT A and B
	Executive functioning	CERAD-NAB: Word-Fluency (phonematic & semantic), TMT B/A ratio;
	Language	CERAD-NAB: BNT
	Visuo-construction	CERAD-NAB: Copy of figures
Self-rated questionnaires	Cognition	SCD-I– participant part
	Depression	GDS-15
	Sleep	PSQI
Informant-rated questionnaires	Cognition	SCD-I – informant part
		IQCODE
	Activities of daily living	IADL
	Behavioral changes	NPI-Q
		MBI-C

BNT: Boston Naming Test; CDR: Clinical Dementia Rating; CDR-SB: Clinical Dementia Rating sum of boxes; CERAD-NAB: Consortium to Establish a Registry for Alzheimer's Disease – Neuropsychological Assessment Battery; GDS: Geriatric Depression Scale; IADL: Instrumental Activities of Daily Living; IQCODE: Informant Questionnaire on Cognitive Decline in the Elderly; MBI-C: Mild Behavioral Impairment Checklist; MMSE: Mini-Mental Status Examination; MoCA: Montreal Cognitive Assessment; NPI-Q: Neuropsychiatric Inventory Questionnaire; PSQI: Pittsburgh Sleep Quality Index; SCD-I: Subjective Cognitive Decline Interview; TMT: Trail Making Test.

### Cognitive, neuropsychiatric, and functional assessments

Neuropsychological assessments were performed either by a neuropsychologist or psychologist in training, who had received onsite instructions and training from the neuropsychologist. The whole test battery, including questionnaires, took approximately 90 min to perform. The participants were first asked about perceived changes in their cognitive abilities in different domains via a structured interview for detailed subjective cognitive decline (SCD-I).^21^ Depressive symptoms and sleep problems were subsequently assessed via the 15-item version ot the Geriatric Depression Scale (GDS)^26^ and the Pittsburgh Sleep Quality Index (PSQI) questionnaire.^27^ As initial cognitive screenings, either the Montreal Cognitive Assessment (MoCA)^28^ or the Mini-Mental State Examination (MMSE)^29^ were performed, depending on the memory clinic center standards. After the initial screening, the Consortium to Establish a Registry for Alzheimer's Disease – Neuropsychological Assessment Battery (CERAD-NAB) was conducted.^30^ The participants’ education level was assessed according to CERAD guidelines. Informant ratings were performed with a relative, spouse or close friend of the participant. Whenever possible, ratings were performed face-to-face or via telephone by a neuropsychologist. If informants were not available for questioning, questionnaires could be filled in by the informant independently. In this case, informants received written instructions. The informant questionnaires included the modified SCD-I,^21^ which inquires about the observed decline. Additionally, cognitive decline was assessed with the Informant Questionnaire on Cognitive Decline in the Elderly (IQCODE).^31,32^ Behavioral changes were assessed with the Neuropsychiatric Inventory (NPI-Q) and the Mild Behavioral Impairment Checklist (MBI-C). The NPI-Q is a self-administered questionnaire that is completed by informants who have regular contact with the individual.^33^ It assesses twelve symptoms: delusions, hallucinations, agitation, dysphoria, anxiety, euphoria, apathy, disinhibition, irritability, aberrant motor activity, nighttime behavioral disturbances, and appetite/eating changes. Each symptom is rated for its presence/absence and if present, also for its severity on a scale from 1–3. The total NPI-Q severity score is obtained by summing the twelve individual scores, yielding a maximum possible score of 36. The MBI-C is also a self-administered questionnaire completed by informants.^34^ It includes five domains with varying numbers of single questions, which are all rated for their presence/absence and if present, for their severity on a scale from 1 to 3: decreased motivation (interest, 6 questions, scoring 0–18), emotional dysregulation (mood, 6 questions, scoring 0–18), impulse dyscontrol (impulsivity, 12 questions, scoring 0–36), social inappropriateness (social, 6 questions, scoring 0–18), and abnormal perception or thought content (psychosis, 5 questions, scoring 0–15). The total MBI-C score is obtained by summing the five domain scores. We used a binary definition of the presence/absence of any NPS. The presence of any NPS was defined either with an NPI-Q severity score >0 or, when the MBI-C was used, if any of the five domains had a score >0. Finally, daily functioning was assessed with the IADL.^24^

### MRI data

We performed structural, quantitative and resting-state functional MRI at baseline according to the protocol of the Alzheimer's Disease Neuroimaging Initiative (ADNI) 3 study at all research sites.^35^ The brain MRI data obtained at the four centers were harmonized via one human MRI phantom. The imaging protocol included the following sequences: structural T1-weighted magnetization-prepared rapid acquisition gradient echo (MPRAGE) for structural analysis, 3D T2-weighted fluid-attenuated inversion recovery (FLAIR) for detecting white matter lesions, T2- and susceptibility-weighted imaging (SWI) to assess cerebral microbleeds, T1-weighted arterial spin labeling (ASL) for detecting metabolic changes, coronal T2-weighted imaging centered on the hippocampus (Carr-Purcell-Meiboom-Gill, CPMG) for hippocampal subfield measurement, and resting-state, i.e., task-free functional MRI (RS-fMRI) for functional analysis.

### Data handling and quality control

All the data were captured through paper-based documents harmonized across the involved centers. In addition, all variables were added to a web-based case report form by each center via the electronic data capture tool REDCap (Yale University, New Haven, CT, USA) REDCap.^36^ Data checking, including ensuring completeness and plausibility, was automatically performed during data entry in REDCap. Offsite monitoring was continuously provided by the central data management unit. Each variable was verified at least by two independent raters, one of which additionally performed a medical review. Inconsistencies were regularly queried for clarification and correction.

### Biological assessments

Detailed standard operating procedures (SOPs) regarding the handling of biological samples are available for each center. The alignment of those procedures was checked regularly both prior to the start of recruitment and under ongoing recruitment regularly to ensure conformity between the centers. Lumbar punctures and venipunctures were performed on the same day between 8:00 and 10:00 am after overnight fasting at the recruiting memory clinics by experienced physicians and nurses.

First, a lumbar puncture was performed. A standard technique using a 22G “atraumatical” spinal needle and a sitting or lying position was applied without prior local anesthesia.^37^ Potentially blood-contaminated CSF was discharged before 8–10 ml of CSF was filled into two polypropylene tubes. After being filled, each tube containing CSF was directly cooled on ice. A total of 350 µL of CSF was then added to 20–30 0.5 ml polypropylene cryotubes (Ratiolab^®^) and stored at −80°C until the assay was performed. The time between lumbar puncture and the storage of CSF samples in the freezer was <20 min, and the CSF was kept cooled on ice during all the described procedures.

Directly after the lumbar puncture, a venipuncture was performed with the participant in a lying position according to standardized techniques. First, serum tubes (containing separation gel) and then plasma tubes (EDTA tubes containing tri-potassium to block the coagulation cascade) were obtained. In total, 50 ml of blood was obtained at baseline and 35 ml was obtained at follow-up. The plasma was placed on ice directly after collection and centrifuged at 6°C for 12 min and 3000 RPM within 15 min after the blood was drawn; then, 350 µL of plasma was added to 20 0.5 ml polypropylene cryotubes (Ratiolab^®^) and stored at −80°C. The time between venipuncture and storage of the plasma samples in the freezer was <30 min, and the plasma was kept on ice during all the described procedures since blood collection. After blood collection, the serum was kept in an upright position for 30 min at room temperature to ensure the correct separation of the serum and the clot. Then, the serum was centrifuged at 6°C for 12 min and 3000 RPM, 350 µL of serum was filled into 20 polypropylene cryotubes (Ratiolab^®^), and stored at −80°C. The time between venipuncture and storage of the serum samples in the freezer was <50 min, and the serum was kept on ice since the start of the centrifugation process 30 min after the blood was drawn.

While several molecular biomarkers have already been measured in the Lausanne-1 center (see detailed list in Supplemental Table 1), we plan to analyze these markers across the entire Omics-AD cohort to generate a harmonized dataset.

### Apolipoprotein E genotyping

*Genotyping.* We extracted DNA from whole blood samples via the QIA Symphony DSP DNA Kit (Qiagen, Hombrechtikon, Switzerland) in a subset of the cohort (n = 206) thus far to determine the apolipoprotein E (*APOE)* alleles (ε2, ε3, ε4, and ε3r). Genotyping for the *APOE* single nucleotide variants rs429358 and rs7412 were performed via the TaqMan assays C___3084793_20 and C___904973_10, respectively (Thermo Fisher Scientific, Waltham, MA, USA). Individuals carrying at least one ε4 allele were classified as *APOE* ε4 carriers.

### CSF ad biomarkers

To date, in 376 participants, the levels of Aβ_42_, total Tau, pTau181, and the Aβ_42_/Aβ_40_ ratio have been measured. We used ELISA (Innotest^®^, Fujirebio Europe, Ghent, Belgium) to measure Aβ_42_, tTau, and pTau181 and immunoassays from IBL International (Hamburg, Germany) to measure the Aβ_42_/Aβ_40_ ratio or the Lumipulse G^®^ (Fujirebio Europe, Ghent, Belgium) automated immunoassay to measure all four markers. All biomarkers were measured according to the manufacturers’ protocols. Amyloid positivity was defined using a cutoff of Aβ_42_/Aβ_40_ < 0.06 or, if the ratio was not available (n = 54), Aβ_42_ < 690 pg/mL according to the laboratory's center cutoff.^38^ The percentage of agreement for amyloid positivity between the Innotest^®^ and Lumipulse^®^ results was calculated for 16 participants, in which both Innotest^®^ and Lumipulse^®^ were performed on the same sample.

### Statistical analysis

We used SPSS (IBM, V.30.0) for the descriptive analysis. We show the means and standard deviations for continuous variables or frequencies and percentages for categorical variables. The normality of continuous variables was assessed via the Shapiro-Wilk test. Group differences in continuous variables were analyzed via t-tests or one-way ANOVA for normally distributed data, and the Mann-Whitney U test or Kruskal-Wallis test for nonparametric data. Categorical variables were compared via Pearson's χ² test. Effect sizes were calculated where appropriate (e.g., Cohens’ d for the t-test, r for Mann-Whitney U test, and Cramér's V for the χ² test) to quantify the magnitude of differences or associations. All tests were two-tailed, with a significance level (α) set at 0.05. For the present analyses, we used a complete-case approach, assuming that missingness was mostly at random. An overview of missing data, including reasons for missingness, is provided in Supplemental Table 2.

### Ethical considerations

The study was conducted in accordance with applicable laws and regulations, including the ethical principles from the Declaration of Helsinki^39^ and the International Conference on Harmonization, Guidelines for Good Clinical Practice.^40^ The study was approved by the local ethics committees in Switzerland: for Lausanne-1 by the canton Vaud (CER-VD No. 171/2013), and for the other three centers (Zurich, Bern, and Lausanne-2) by the canton Zurich (BASEC No. 2021-00305). Written informed consent was obtained from all participants prior to inclusion, including separate consent for the storage of biological materials in a biobank.

## Results

**Figure 2.**
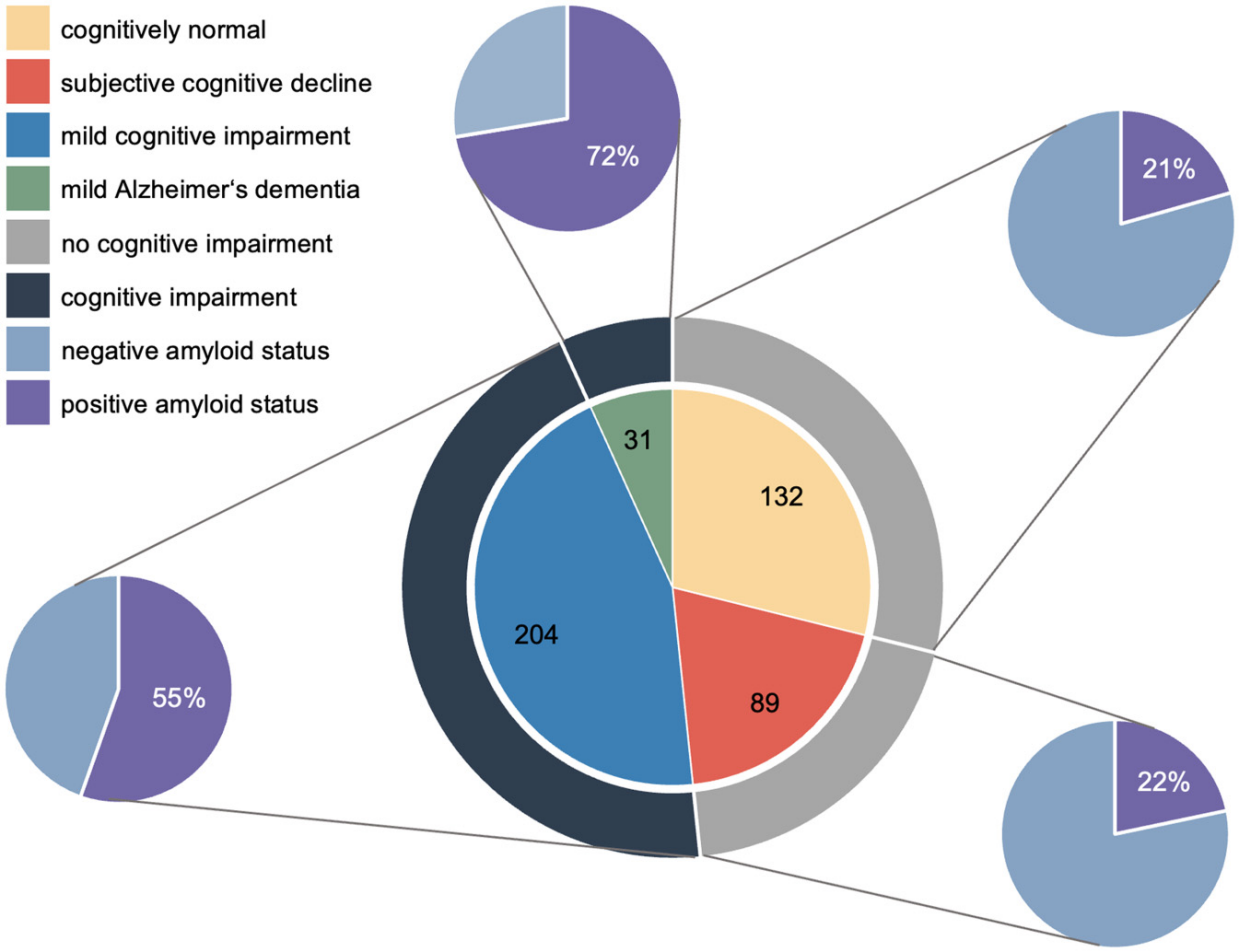
Diagnosis of the study participants in the Omics-AD cohort study including the amyloid status.

All included participants and their obtained data until the end of July 2025 were considered for this analysis. A total of 456 participants in the four centers (217 in Lausanne-1, 155 in Zurich, 61 in Lausanne-2, and 23 in Bern) were included in this analysis. Among the included participants, 221 (48.5%) were cognitively unimpaired (89 with SCD and 132 with normal cognition and no cognitive complaints), and 235 (51.5%) were cognitively impaired (204 with MCI and 31 with mild clinical AD dementia), as shown in Figure 2.

The detailed cohort characteristics per clinical diagnosis are shown in Table 2. At inclusion, participants with cognitive impairment (MCI or mild clinical AD dementia) were generally older (73.3 ± 6.4 years, versus 68.9 ± 6.3 years, t(440) = 7.25, p < 0.001, Cohen's d = 0.69), had fewer years of education (13.2 ± 3.6 years versus 14.1 ± 3.1 years, U = 20811.50, Z = -2.58, p = 0.010, r = -0.12), presented more severe behavioral symptoms (NPI-Q severity score 3.0 ± 4.2 versus 0.9 ± 1.8, U = 20079.0, Z = -5.95, p < 0.001, r = -0.32) and more often had concomitant cardiovascular conditions – hypertension (41.6% versus 28.3%, χ²(1, N = 424) = 8.18, p = 0.004, Cramer's V = 0.14), hypercholesterinemia (43.6% versus 26.5%, χ²(1, N = 416) = 13.23, p < 0.001, Cramer's V = 0.18), and diabetes (10.4% versus 4.1%, χ²(1, N = 396) = 6.16, p = 0.016, Cramer's V = 0.12) – when compared with cognitively unimpaired participants (NC and SCD).

**Table 2.** Characteristics of the study participants.

	Total n = 456	NC n = 132	SCD n = 89	MCI n = 204	dementia n = 31	p
**Demographic data**						
female, n (%)	252 (55.1)	79 (59.8)	52 (58.4)	101 (49.3)	20 (64.5)	0.137^a^
age, mean ± s.d.	71.2 ± 6.7	68.6 ± 6.3	69.7 ± 6.1	73.0 ± 6.4	75.2 ± 5.8	<0.001^b^
education years, mean ± s.d.	13.6 ± 3.4	13.9 ± 2.7	14.5 ± 3.3	13.4 ± 3.7	12.1 ± 2.8	0.002^c^
BMI, mean ± s.d.	24.9 ± 4.0	24.6 ± 3.9	24.9 ± 3.8	25.2 ± 4.3	23.8 ± 3.5	0.281^c^
**Cognitive and functional parameters, mean** **±** **s.d.**						
MoCA	23.4 ± 4.9	26.0 ± 2.6	26.6 ± 2.6	22.0 ± 5.0	15.0 ± 4.7	<0.001^c^
CDR-SB	1.0 ± 1.7	0.4 ± 0.1	0.5 ± 0.1	1.3 ± 1.0	5.9 ± 1.9	<0.001^c^
IQCODE score	3.3 ± 0.5	3.0 ± 0.4	3.1 ± 0.3	3.5 ± 0.5	4.1 ± 0.5	<0.001^c^
IADL	7.4 ± 1.3	7.9 ± 0.4	7.8 ± 0.5	7.2 ± 1.4	5.1 ± 1.8	<0.001^c^
**Behavioral parameters (including minimum and maximum of each scale), mean** **±** **s.d.**						
GDS-15 (0–15)	1.7 ± 2.6	0.8 ± 1.4	1.8 ± 3.9	2.1 ± 2.1	2.3 ± 1.6	<0.001^c^
NPI-Q severity score (0–36)	2.0 ± 3.4	1.0 ± 2.0	0.8 ± 1.3	2.8 ± 4.0	5.9 ± 5.3	<0.001^c^
MBI-C total score (0–105)	4.1 ± 7.0	1.5 ± 3.4	1.6 ± 3.6	6.0 ± 7.7	18.9 ± 10.7	<0.001^c^
MBI-C interest (0–18)	1.1 ± 2.3	0.3 ± 0.8	0.5 ± 1.5	1.6 ± 2.3	5.7 ± 4.8	<0.001^c^
MBI-C mood (0–18)	1.2 ± 2.4	0.3 ± 0.8	0.4 ± 1.4	1.7 ± 2.4	6.8 ± 4.3	<0.001^c^
MBI-C impulsivity (0–36)	1.4 ± 2.9	0.7 ± 2.2	0.5 ± 1.1	2.1 ± 3.4	4.8 ± 5.2	<0.001^c^
MBI-C social (0–18)	0.3 ± 0.9	0.2 ± 0.7	0.2 ± 0.6	0.5 ± 1.0	1.0 ± 1.8	0.015^c^
MBI-C psychosis (0–15)	0.1 ± 0.6	0.1 ± 0.2	0.1 ± 0.4	0.2 ± 0.7	0.7 ± 1.0	<0.001^c^
**History of medical conditions, n (%)**						
Hypercholesterolemia	152 (35.4)	37 (29.4)	19 (22.9)	84 (42.9)	12 (50.0)	0.002 ^a^
Hypertension	154 (35.2)	33 (25.8)	27 (32.1)	81 (40.5)	13 (50.0)	0.016 ^a^
Depression	92 (21.2)	21 (16.5)	17 (20.2)	45 (23.2)	9 (32.1)	0.244 ^a^
Neurological	59 (13.4)	19 (15.1)	12 (14.6)	22 (10.9)	6 (19.4)	0.483 ^a^
Cranial trauma	61 (13.8)	14 (11.1)	22 (26.8)	24 (11.9)	1 (3.2)	0.001 ^a^
Diabetes	33 (7.5)	6 (4.7)	3 (3.6)	21 (10.3)	3 (10.7)	0.107 ^a^

^a^ Chi square test, ^b^One-way ANOVA test, ^c^Kruskal-Wallis test; p-value indicates the overall statistical significance of differences among the four groups.

BMI: body mass index; CDR-SB: Clinical Dementia Rating sum-of-boxes; GDS: Geriatric Depression Scale 15-item version; IADL: Instrumental Activities of Daily Living; IQCODE: Informant Questionnaire on Cognitive Decline in the Elderly; MMSE: Mini-Mental Status Examination; MBI-C: Mild Behavioral Impairment Checklist; MoCA: Montreal Cognitive Assessment; n: number; NPI-Q: Neuropsychiatric Inventory Questionnaire.

**Figure 3.**
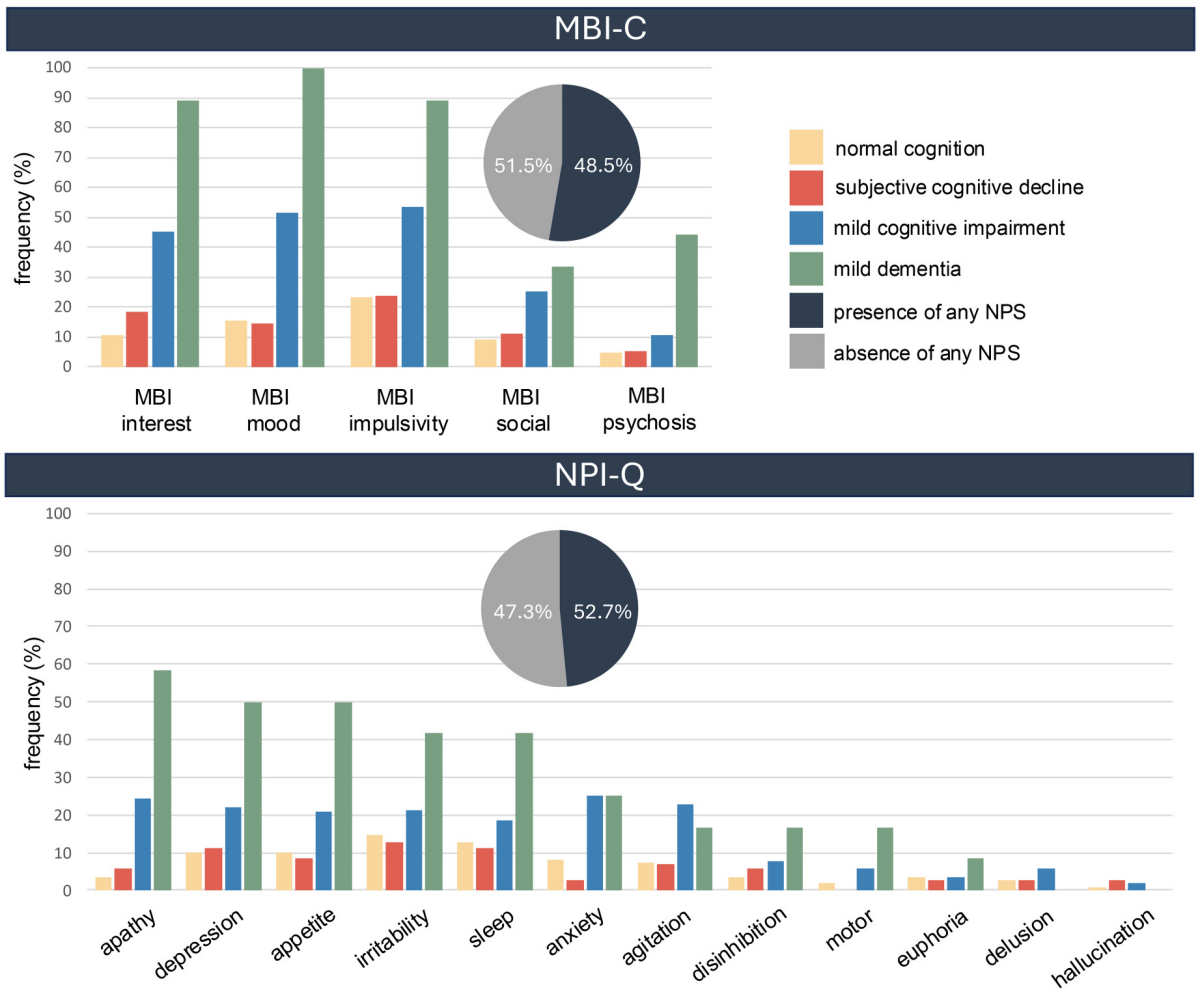
Frequencies of different neuropsychiatric symptoms measured by the NPI-Q and MBI-C. The NPI-Q was administered to n = 361 participants and the MBI-C was administered to n = 224 participants. The presence of any NPS was defined either with an NPI-Q severity score >0 or, when the MBI-C was used, if any of the five domains had a score >0. MBI-C, Mild Behavioural Impairment Checklist; NPI-Q, Neuropsychiatric Inventory Questionnaire.

Approximately half of the included participants experienced any NPS (48.5% as measured by the NPI-Q and 52.7% as measured by the MBI-C). Subgroup analysis for the four diagnosis groups revealed that 33.6% of NC, 39.4% of SCD, 59.9% of MCI, and 75.0% of dementia patients experienced any NPS based on the NPI-Q or 32.3% of NC, 36.4% of SCD, 72.3% of MCI, and 100% of dementia patients based on the MBI-C, respectively. Using a validated cutoff of total MBI-C score > 7 to define the presence of MBI,^41^ we found a prevalence of 18.0% in all participants (6.2% of NC, 5.5% of SCD, 26.9% of MCI, and 88.9% of dementia patients). The most common behavioral symptoms measured by the NPI-Q were irritability (18%) and depression (17%). The most frequent MBI-C domains were impulsivity (39%) and mood dysregulation (34%). The detailed frequency of behavioral symptoms is shown in Figure 3. We collected information on informant relationships in two centers (Lausanne-2 and Zurich). Informants were most often partners (n = 114, 57.0%), followed by children (n = 43, 21.5%), close friends (n = 20, 10.0%), siblings (n = 10, 5.0%), and other types of relationships (n = 4, 2.0%). For nine participants, informants declined participation, and their relationship could not be documented.

### Availability

Clinical and neuropsychological data as well as blood samples were obtained for all included participants, CSF samples in 93.2% (n = 426), MRI data in 73.3% (n = 335), and proxy questionnaires in 91.2% (n = 417) of the cases. In the already concluded Lausanne-1 study, 69.1% of the participants had at least one follow-up (FU) visit, with a mean FU time of 17.7 ± 7.2 months for the first available FU visit. The reasons for dropping out at the Lausanne-1 center were the end of the study (n = 40), a decline in further participation in FU visits (n = 13), participants’ death (n = 6), lost contact (n = 4), moving (n = 2), or unknown (n = 3). Among the planned FU visits in the three centers with ongoing recruitment (Lausanne-2, Zurich, Bern) 84.5% have been performed thus far. Details on dropout rates are not available, as FU visits are ongoing, and participants may still perform FU visits at a later timepoint than planned. Details on missing data are shown in Supplemental Table 2.

### Concomitant medication

The detailed intake of medication at all visits, including phytotherapies, was obtained. Only participants with stable medications over the last three months were included. The compounds most frequently taken at baseline were supplements, including phytotherapies and vitamins, antihypertensives, and statins. A detailed list of concomitant medications is shown in Supplemental Table 3.

### Amyloid pathology

Supplemental Tables 4 and 5 show CSF levels for each clinical diagnosis, using Innotest^®^/IBL^®^ and Lumipulse^®^. In total, 41% (n = 155) of the participants had a positive amyloid status (20.6% (n = 22) in the NC group, 21.7% (n = 15) in the SCD group, 55.4% (n = 97) in the MCI group, and 72.4% (n = 21) in the mild clinical AD dementia group), as shown in Figure 2. In 16 participants both Innotest^®^ and Lumipulse^®^ were measured. The amyloid status for all 16 participants matched between both methods. Spearman analysis of the correlations between Innotest^®^/IBL^®^ and Lumipulse^®^ (n = 16) revealed strong correlations between both methods: Aβ_42_ r = 0.826, p < 0.001; Aβ_42_/Aβ_40_ r = 0.673, p < 0.008; tTau r = 0.943, p < 0.001; pTau181 r = 0.959, p < 0.001; and pTau181/Aβ_42_ r = 0.926, p < 0.001.

## Discussion

The Omics-AD study is a multi-centric, longitudinal brain aging cohort study that currently includes more than 450 older individuals in Switzerland. Clinical and neuropsychological data were collected via standardized and widely used assessment methods in the field.^42^ Additionally, extensive molecular analysis data from paired blood and CSF samples along with MRI features were obtained, enabling high-quality single omics and multi-omics analysis. NPS were present in approximately half of the participants, allowing for the comprehensive investigation of alterations in NPS in general and the most common single symptoms in particular. Amyloid status was derived based on CSF amyloid. Follow-up visits were performed for 85% of the participants thus far.

To this day, we included 456 participants, with a well-balanced distribution of 48% of the participants being cognitively unimpaired (29% NC, 20% SCD) and 52% cognitively impaired (45% MCI, 7% mild clinical AD dementia). Within the cognitively unimpaired group, we further differentiated between individuals with SCD and those with no cognitive complaints (NC) allowing for a nuanced analysis of preclinical AD stages. We included a large proportion of participants with MCI with the aim of investigating the early clinical stages of AD. Other AD research cohorts, such as the ADNI cohort, included higher proportions of cognitively impaired participants, especially dementia patients (17%),^43^ whereas the single-center Amsterdam Dementia cohort included 29% of participants with clinical AD dementia.^44^ Hence, our study stands out for its substantial number and large proportion of cognitively unimpaired participants. Importantly, we obtained CSF samples from the vast majority of these participants (93%), providing a unique opportunity to investigate early molecular changes in association with AD CSF biomarkers before clinical symptoms emerge.

In our cohort, 48% of the participants experienced NPS, with irritability and depression being the most common single symptoms (18% and 17%, respectively). NPS were more common with greater cognitive impairment. These findings are consistent with those of previous reports, which estimated the NPS prevalence to be 35–75% in patients with MCI^45,46^ and up to 97% in dementia patients.^47^ For example, in a study on proteomic profiles of NPS in the ADNI cohort, 46% exhibited any NPS, with irritability (30%) and depression (23%) being the most common symptoms.^20^

We assessed NPS using two instruments: the NPI-Q and the MBI-C. While the NPI-Q remains the most widely used tool for NPS assessment, the MBI-C has recently gained traction. It was developed specifically to identify the onset of NPS in later life, which is related to a greater risk for developing cognitive decline and dementia.^48^ Importantly, our cohort, characterized by a high proportion of cognitively unimpaired participants with and without underlying AD pathology, offers a unique opportunity to investigate the contribution of the NPS to disease progression in the early clinical stages of AD.

Based on CSF analysis, we determined the amyloid status of 376 participants thus far. A total of 41% of all participants had a positive amyloid status (21% in NC, 22% in SCD, 55% in MCI, 72% in clinical AD dementia). The prevalence of amyloid pathology across the single diagnosis groups in our cohort was very similar to findings from the Amyloid Biomarker Study (ABS), an ongoing, worldwide data-pooling initiative that started in 2013 and includes more than 85 cohorts thus far.^49^ Among the participants in the ABS, 21–28% of NC, 21–33% of SCD, 46–56% of MCI, and approximately 79% of clinical AD dementia patients had a positive amyloid status.^50^

CSF AD biomarkers were measured via either the Innotest^®^ or Lumipulse^®^ platforms, except for 16 samples, which were measured with both methods. Strong correlations were observed for Aβ_42_, Aβ_42_/Aβ_40_, tTau, and pTau181 when the two platforms were compared, and the amyloid status was identical in all 16 paired samples. This high concordance between Innotest^®^ and Lumipulse^®^ has been reported previously.^51,52^ However, the number of paired measurements in our study was relatively small; therefore, Lumipulse^®^ analyses will be repeated in a larger sample set.

Using the data from the Lausanne-1 center, we previously performed different omics analyses to detect pathophysiological changes related to AD and associated symptoms such as NPS. By applying an integrative multi-omics approach, we reported novel molecules and pathways associated with AD pathology, showing the potential of our cohort to discover biological alterations relevant to AD.^9,16^ Furthermore, we applied single omics to explore neuroinflammatory molecules and the proteome alterations related to NPS in CSF and blood.^53–55^

Further planned analyses include single omics, such as proteomics, metabolomics, and lipidomics in blood plasma or serum and CSF in the whole cohort. The findings will be integrated into multi-omics models and related to available clinical and imaging data. Using data-driven approaches we expect to gain a better understanding of the pathophysiology of AD and AD-related symptoms including cognitive decline and the persistence of NPS over time, as well as to identify new biomarker candidates. Furthermore, we intend to perform targeted analysis of single molecules and selected molecule panels to validate and extend previous findings, such as inflammatory alterations in NPS.^54,55^

Major strengths of this cohort study are its multicentric study design along with the SOP harmonization between the four centers that used the same study protocol, reducing variability related to data and biological sample collection and assessment methods. Additionally, CSF was obtained from a large proportion of the participants, including more than two hundred cognitively unimpaired participants. We were able to determine both the clinical diagnosis and biomarker confirmation of AD pathology in almost all participants. The well-balanced sex distribution in all clinical subgroups will also allow sex-specific alterations to be addressed. Real-life data considering different possible interfering factors, such as medication and comorbid diseases, are available.

However, this study also has several limitations. We included only participants at early clinical stages, not allowing us to investigate more severe disease stages such as moderate or severe dementia. Additionally, individuals with moderate to severe NPS interfering with cognition were excluded. Some NPS, such as delusions or hallucinations, were very rare in this cohort, preventing the investigation of those symptoms in more detail. On the other hand, we focused on early AD stages and were able to include a high number of cognitively unimpaired individuals, enabling us to capture preclinical pathophysiological mechanisms involved in AD. Therefore, we expect this cohort and the upcoming results to be particularly relevant for memory clinic patients, who in most cases have SCD, MCI, or mild dementia. We also acknowledge that the use of complete-case analysis may introduce bias if the missingness mechanism was not completely at random. Depending on future completion rates of FU visits, missing data will be investigated more precisely, as NPS may significantly affect completion rates.

### Conclusions

Omics-AD is a large, multi-centric, longitudinal, and well-characterized brain aging cohort unique in Switzerland. This cohort includes multiple data levels and allows for the integrative analysis of different omics datasets with a focus on the preclinical and early stages of AD. This study is expected to offer comprehensive insights into the pathophysiology of AD and AD-related symptoms, including both cognitive decline and NPS, and to identify potential treatment targets and new diagnostic and prognostic biomarkers.

## Supplemental Material

sj-docx-1-alz-10.1177_13872877251401159 - Supplemental material for Omics-AD—A multimodal biomarker study on cognitive decline and neuropsychiatric symptoms: Design and cohort characteristics Supplemental material, sj-docx-1-alz-10.1177_13872877251401159 for Omics-AD—A multimodal biomarker study on cognitive decline and neuropsychiatric symptoms: Design and cohort characteristics by Miriam Rabl, Leonardo Zullo, Jelena Wehrli, Karolin Hössel, Piotr Lewczuk, Iliya Petkov Peyneshki, Erich Seifritz, Stefan Klöppel, Armin von Gunten and Julius Popp in Journal of Alzheimer's Disease
