# Supplementary material for: Omics-AD—A multimodal biomarker study on cognitive decline and neuropsychiatric symptoms: Design and cohort characteristics
Source: J Alzheimers Dis. 2025 Dec 4;109(2):889–901. doi: 10.1177/13872877251401159 (PMC12775174; doi:10.1177/13872877251401159)
Supplement: sj-docx-1-alz-10.1177_13872877251401159 - Supplemental material for Omics-AD—A multimodal biomarker study on cognitive decline and neuropsychiatric symptoms: Design and cohort characteristics [file sj-docx-1-alz-10.1177_13872877251401159.docx]

**Supplemental Material**

**Omics-AD—A multimodal biomarker study on cognitive decline and neuropsychiatric symptoms: Design and cohort characteristics**

**Supplemental Table 1.** Overview of already available analytes of the Lausanne-1 center within the Omics-AD cohort study

| **Biomarker** | **Specimen** | **Reference, if available** |
| --- | --- | --- |
| Cholesterol and cholesterol metabolites | CSF | ^1^ |
| Cortisol and Dehydroepiandrosterone Sulfate | CSF | ^2,3^ |
| Matrix-Metalloproteinase 10 | CSF |  |
| One-carbon metabolism: | CSF | ^4^ |
| Neuroinflammatory proteins | CSF | ^5,6^ |
| Ionomics | CSF |  |
| Cytokines | CSF |  |
| Metabonomics | CSF |  |
| Lipidomics | CSF | ^7^ |
| Fattc acids | CSF |  |
| Proteomics | CSF | ^7-9^ |
| Albumin ratio for blood-brain barrier impairment | CSF/serum | ^4^ |
| Neuroregulatory proteins | serum | ^10^ |
| Nutrients and vitamins | serum |  |
| Ionomics | serum | ^11^ |
| Neuroinflammatory proteins | serum | ^4,6^ |
| Fatty acids | serum |  |
| Amino acids | serum |  |
| Metabolomics | serum |  |
| Phosphorylated Tau at threonine 181 | plasma | ^12,13^ |
| Neurofilament light chain | plasma | ^12,13^ |
| Glial fibrillary acid protein | plasma | ^13^ |
| One-carbon metabolism | plasma | ^14,15^ |
| Cholesterol metabolism and bile acids | plasma |  |
| Lipidomics | plasma |  |
| Proteomics | plasma | ^16^ |
| *APOE* genotyping | blood |  |
| Micro RNA | blood |  |

**References**

1. Jahn T, Clark C, Kerksiek A, et al. Cholesterol metabolites and plant sterols in cerebrospinal fluid are associated with Alzheimer's cerebral pathology and clinical disease progression. *J Steroid Biochem Mol Biol* 2021; 205: 105785.

2. Ouanes S, Clark C, Richiardi J, et al. Cerebrospinal fluid cortisol and dehydroepiandrosterone sulfate, Alzheimer's disease pathology, and cognitive decline. *Front Aging Neurosci* 2022; 14: 892754.

3. Ouanes S, Rabl M, Clark C, et al. Persisting neuropsychiatric symptoms, Alzheimer's disease, and cerebrospinal fluid cortisol and dehydroepiandrosterone sulfate. *Alzheimers Res Ther* 2022; 14: 190.

4. Bowman GL, Dayon L, Kirkland R, et al. Blood-brain barrier breakdown, neuroinflammation, and cognitive decline in older adults. *Alzheimers Dement* 2018; 14: 1640-1650.

5. Popp J, Oikonomidi A, Tautvydaitė D, et al. Markers of neuroinflammation associated with Alzheimer's disease pathology in older adults. *Brain Behav Immun* 2017; 62: 203-211.

6. Clark C, Richiardi J, Maréchal B, et al. Systemic and central nervous system neuroinflammatory signatures of neuropsychiatric symptoms and related cognitive decline in older people. *J Neuroinflammation* 2022; 19: 127.

7. Clark C, Dayon L, Masoodi M, et al. An integrative multi-omics approach reveals new central nervous system pathway alterations in Alzheimer's disease. *Alzheimers Res Ther* 2021; 13: 71.

8. Dayon L, Cominetti O, Wojcik J, et al. Proteomes of paired human cerebrospinal fluid and plasma: relation to blood-brain barrier permeability in older adults. *J Proteome Res* 2019; 18: 1162-1174.

9. Mroczek M, Clark C, Dayon L, et al. Cerebrospinal fluid proteome alterations associated with neuropsychiatric symptoms in cognitive decline and Alzheimer's disease. *Cells* 2022; 11: 1030.

10. Menne F, Schipke CG, Clark C, et al. Long-term stability and age-dependence of six regulatory serum proteins. *Biomark Med* 2022; 16: 511-521.

11. Konz T, Migliavacca E, Dayon L, et al. ICP-MS/MS-based ionomics: a validated methodology to investigate the biological variability of the human ionome. *J Proteome Res* 2017; 16: 2080-2090.

12. Clark C, Lewczuk P, Kornhuber J, et al. Plasma neurofilament light and phosphorylated tau 181 as biomarkers of Alzheimer's disease pathology and clinical disease progression. *Alzheimers Res Ther* 2021; 13: 65.

13. Rabl M, Zullo L, Lewczuk P, et al. Plasma neurofilament light, glial fibrillary acid protein, and phosphorylated tau 181 as biomarkers for neuropsychiatric symptoms and related clinical disease progression. *Alzheimers Res Ther* 2024; 16: 165.

14. Guiraud SP, Montoliu I, Da Silva L, et al. High-throughput and simultaneous quantitative analysis of homocysteine-methionine cycle metabolites and co-factors in blood plasma and cerebrospinal fluid by isotope dilution LC-MS/MS. *Anal Bioanal Chem* 2017; 409: 295-305.

15. Dayon L, Guiraud SP, Corthésy J, et al. One-carbon metabolism, cognitive impairment and CSF measures of Alzheimer pathology: homocysteine and beyond. *Alzheimers Res Ther* 2017; 9: 43.

16. Rabl M, Clark C, Dayon L, et al. Blood plasma protein profiles of neuropsychiatric symptoms and related cognitive decline in older people. *J Neurochem* 2023; 164: 242-254.

| **Supplemental Table 2.** Overview of missing data in the Omics-AD cohort | | | |
| --- | --- | --- | --- |
| **Instrument** | **Availability, n (%)** | **Reason for missingness (n)** |  |
| CSF | 426 (93.2) | LP failed/denied (30) |  |
| CSF core AD biomarker | 378/426 (88.7) | not yet measured (48) |  |
| MRI | 335 (73.3) | memory-clinic patients performed MRI with a separate MRI scanner through the hospital routine (121) |  |
| History of medical conditions | 447 (98.0) | missing at random (9) |  |
| History of phytotherapies | 239 (52.4) | not obtained in Lausanne-1 (217) |  |
| BMI | 439 (96.3) | missing at random (17) |  |
| informant relationship | 200 (43.9) | not obtained in Lausane-1 (217) and Bern (23)  missing at random (16) |  |
| IADL | 391 (85.7) | not obtained in Lausanne-1 (55)  missing at random (10) |  |
| IQCODE | 379 (83.1) | not obtained in Lausanne-1 (62)  missing at random (15) |  |
| NPI-Q | 361 (79.2) | not obtained in Lausanne-1 (80)  missing at random (15) |  |
| MBI-C | 224 (49.1) | not obtained in Lausanne-1 (217)  missing at random (15) |  |
| GDS | 236 (51.8) | not obtained in Lausanne-1 (217)  missing at random (3) |  |

**Supplemental Table 3.** Detailed medication intake at inclusion

| **Medication intake at inclusion** |  | **n (%)** |
| --- | --- | --- |
| Supplements incl. vitamins |  | 112 (65.5)* |
| Anti-Hypertensives |  | 143 (32.1) |
| Statins |  | 130 (29.2) |
| Aspirin |  | 96 (21.5) |
| Antidepressants |  | 84 (19.2) |
| Ginkgo |  | 42 (9.4) |
| Anti-inflammatory |  | 41 (9.2) |
| Anti-Diabetic |  | 35 (8.3) |
| Benzodiazepine |  | 23 (5.2) |
| Neuroleptics |  | 13 (2.9) |

* information not available for Lausanne-1

**Supplemental Table 4.** CSF levels (pg/mL) of AD biomarkers measured with Lumipulse® technology

| **Lumipulse**®**,**  **mean (s.d.)** | **Total**  n = 168 | **NC**  n = 42 | **SCD**  n = 42 | **MCI**  n = 76 | **AD**  n = 8 | **p** |
| --- | --- | --- | --- | --- | --- | --- |
| Aβ_42_ | 778.7 ± 364.3 | 871.4 ± 354.8 | 882.6 ± 328.7 | 688.7 ± 365.5 | 602.3 ± 352.7 | 0.001 ^a^ |
| total Tau | 366.2 ± 193.3 | 311.1 ±178.9 | 290.1 ± 116.1 | 411.9 ± 193.4 | 581.5 ± 296.5 | <0.001 ^a^ |
| pTau181 | 44.4 ± 25.4 | 34.6 ±14.5 | 35.9 ± 16.0 | 51.5 ± 27.9 | 73.7 ± 42.8 | <0.001 ^a^ |

^a^Kruskal-Wallis test; p-value indicates the overall statistical significance of differences among the four groups.

**Supplemental Table 5.** CSF levels (pg/mL) of AD biomarkers measured with Innotest® technology

| **Innotest**®**,**  **mean (s.d.)** | **Total**  n = 224 | **NC**  n = 64 | **SCD**  n = 27 | **MCI**  n = 110 | **AD**  n = 24 | **p** |
| --- | --- | --- | --- | --- | --- | --- |
| Aβ_42_ | 864.0 ± 308.1 | 1025.5 ± 266.2 | 1011.6 ± 277.0 | 768.3 ± 265.1 | 705.9 ± 377.4 | <0.001 ^a^ |
| total Tau | 427.7 ± 298.7 | 299.1 ± 177.5 | 343.4 ± 228.1 | 502.9 ± 337.3 | 520.9 ± 306.6 | <0.001 ^a^ |
| pTau181 | 66.7 ± 34.6 | 54.4 ± 20.9 | 60.2 ± 25.6 | 71.9 ± 32.8 | 82.1 ± 61.1 | <0.001 ^a^ |

^a^Kruskal-Wallis test; p-value indicates the overall statistical significance of differences among the four groups.
